# Value of Drains in Soft-Tissue Tumour Surgery: A Specialist Regional Centre Experience

**DOI:** 10.7759/cureus.32259

**Published:** 2022-12-06

**Authors:** Omar El-Omar, Umair Akbani, Abdelrahim Abdelrahman, Amir R Akbari, Umar N Said, Omar M Ismail, Cristian Butaliu, Ashok S Paul

**Affiliations:** 1 The Regional Sarcoma Centre, Manchester Royal Infirmary, Manchester, GBR; 2 Obstetrics and Gynaecology, King’s Mill Hospital, Mansfield, GBR; 3 Trauma & Orthopaedics, Huddersfield Royal Infirmary, Huddersfield, GBR; 4 Trauma & Orthopaedics, Royal Lancaster Infirmary, Lancaster, GBR

**Keywords:** soft-tissue tumour, infection, drain, compression bandage, sarcoma

## Abstract

Background

The mainstay of therapy in most soft-tissue tumours (STTs) is excision. However, this often results in blood/extracellular fluid collection within large dead spaces necessitating the use of surgical drains. Whether meticulous attention to haemostasis, careful closure of dead space, and use of compression bandage obviates the need for drains was investigated. This study aimed to compare postoperative outcomes in patients undergoing surgery for STTs with and without the use of drains.

Methodology

A retrospective analysis of patients undergoing STT surgery over five years was undertaken using a regional STT specialist service database. Patients were stratified into the following two groups: compression bandage alone (CB) versus compression bandage with drain (CBD). The chi-square test was used to examine associations with infection, seroma, and haematoma, while the unpaired t-test was used for associations with hospital stay and time to wound healing. The unpaired t-test with Bonferroni correction was used to account for tumour dimensions across both groups.

Results

A total of 81 CB and 25 CBD patients were included. The mean hospital stay was significantly lower in CB compared to CBD (4.9 days, SD = 8.574 vs. 9.8 days, SD = 7.647, p = 0.0125). None of the other variables was significantly different between the two groups, including infection (21.3% vs. 24.0%, p = 0.7804), seroma (25.0% vs. 36.0%, p = 0.2865), haematoma (0.026% vs. 2.0%, p = 0.2325), and time to wound healing (55.8 days, SD = 63.59 vs. 42.3 days, SD = 58.88, p = 0.3648).

Conclusions

Our findings suggest that the use of drains in patients undergoing STT tumour surgery lengthens hospital stay without reducing the incidence of postoperative complications/time to wound healing. A larger, prospective trial is needed.

## Introduction

Soft-tissue sarcomas are a rare and heterogeneous group of over 60 different neoplasms of mesenchymal origin. Due to their propensity for metastasis, soft-tissue sarcomas represent approximately 2% of cancer-related deaths. Accounting for <1% of all neoplasms [1], sarcoma has a prevalence of around two to four people per 100,000 population [2].

Historically, for soft-tissue tumours (STTs) presenting on the limbs, the cornerstone for management was amputation. Advances in surgical technique and multimodality therapies including chemotherapy and radiotherapy have allowed for limb-preserving surgery to become much more common [2]. With this approach, the tumour is excised, and the wound is sealed. Once removed, a cavity remains in place of the tumour, known as dead space. Closure of this dead space and maintaining thorough haemostasis are key to improving postoperative morbidity [3]. A compression bandage is also applied to minimise swelling. However, blood or extracellular fluid may inevitably collect within the wound. Traditionally, surgical drains have been used in the resection of these tumours as a routine. Drains can also be used with amputations. This allows for drainage of the blood or extracellular fluid that has gathered, thereby reducing pressure on the surgical site and nearby structures. This enhances wound perfusion and healing, thereby decreasing the likelihood of postoperative local wound complications [4]. However, the use of drains may not be entirely beneficial. Insertion of this foreign body may increase the likelihood of local infection. Drains also remain in place for several days or weeks until satisfactory drainage is achieved. Patients tend to remain in the hospital during this time, and this further increases the possibility of cross-infection. As such, depending on the risk-benefit ratio, drains may not be used. However, whether the combination of adequate perioperative haemostasis using Surgicel, closure of the dead space to the greatest possible extent, and compression bandage alone eliminates the need for drains requires further study. The use of tranexamic acid during surgery to decrease postoperative bleeding also needs further investigation.

To our knowledge, there are no studies comparing outcomes in limb STT patients in whom surgical drains were inserted versus those without. We aim to explore whether the use of surgical drains had any effect on the length of hospital stay, time to wound healing, surgical site infection (SSI), and development of seroma and haematoma.

This article was previously presented as a meeting abstract at the 2020 BOOS Annual Scientific Meeting on November 13, 2020.

## Materials and methods

A total of 106 STT patients who underwent surgery over a five-year period (2015-2020) at a regional STT specialist service were analysed. Patients were categorised into the following two groups: compression bandage alone (CB) and compression bandage with drain (CBD). Data were collected on a total of 81 CB and 25 CBD patients. These included various postoperative complications, defined as seroma (a collection of fluid within the wound as identified clinically or via radiological imaging, without infection), haematoma (localised bleeding as identified clinically or via radiological imaging, without infection), and SSI (a wound with positive cultures, purulent drainage, or designated an infection by the medical or surgical team). We further analysed data observing the length of hospital stay and time to wound healing. Associations with seroma, haematoma, and SSI were evaluated using the chi-square test, while the unpaired t-test was used for associations with hospital stay and time to wound healing.

Tumour length, width, and depth were recorded in millimetres utilising radiological reports from the electronic database. A mean was calculated with 95% confidence intervals for each dimension. The unpaired t-test with Bonferroni correction was performed to assess statistical significance.

Further data collected included age, sex, soft-tissue tumour type, tumour location, development of metastasis, tumour recurrence, and mortality. Patients were followed up for a minimum of 12 months after the primary procedure or until their death.

## Results

**Figure 1:**
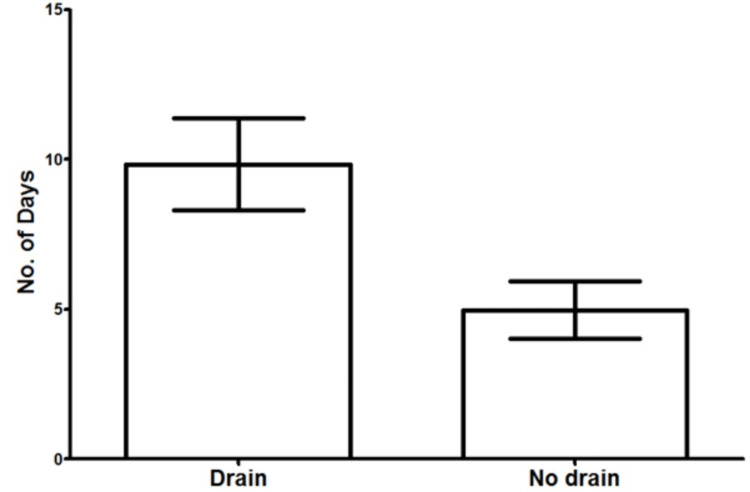
The mean hospital stay in the CB versus CBD group. The mean hospital stay in patients without drains was significantly lower than patients with drains: 4.98 days versus 9.84 days, respectively, p = 0.0125. CB = compression bandage alone; CBD = compression bandage with drain

**Figure 2:**
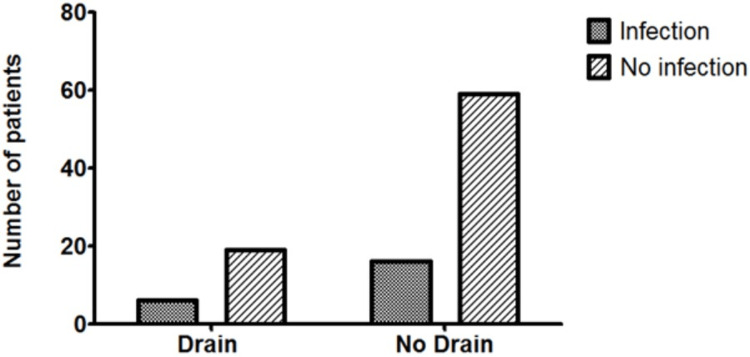
The incidence of SSI in the CB versus CBD group. The presence of a drain did not significantly affect the incidence of SSI in patients with drains versus those without: 21.3% versus 24.0%, respectively, p = 0.7804. CB = compression bandage alone; CBD = compression bandage with drain; SSI = surgical site infection

**Figure 3:**
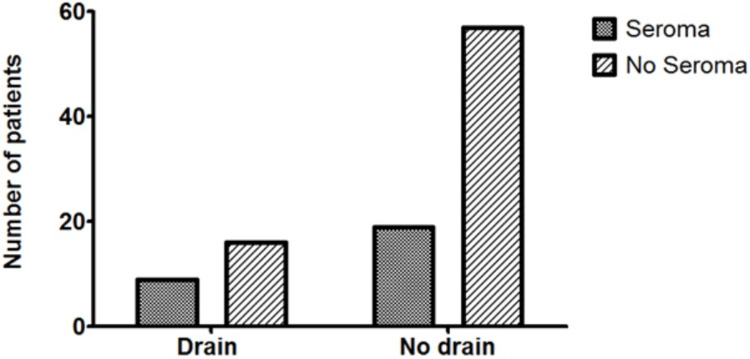
The incidence of seroma in the CB versus CBD group. The presence of a drain did not significantly affect the incidence of seroma in patients with drains versus those without: 25.0% versus 36.0%, respectively, p = 0.2865. CB = compression bandage alone; CBD = compression bandage with drain

**Figure 4:**
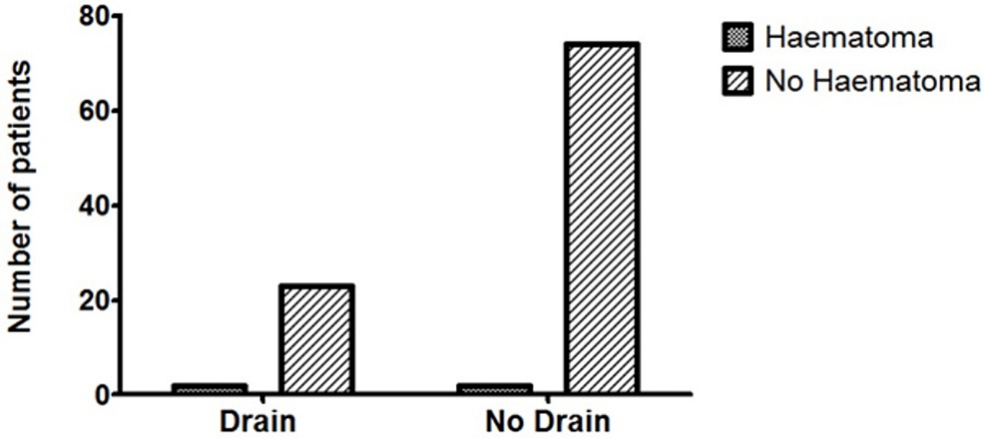
The incidence of haematoma in the CB versus CBD group. The presence of a drain did not significantly affect the incidence of haematoma in patients with drains versus those without: 0.026% versus 8.0%, respectively, p = 0.2325. CB = compression bandage alone; CBD = compression bandage with drain

**Figure 5:**
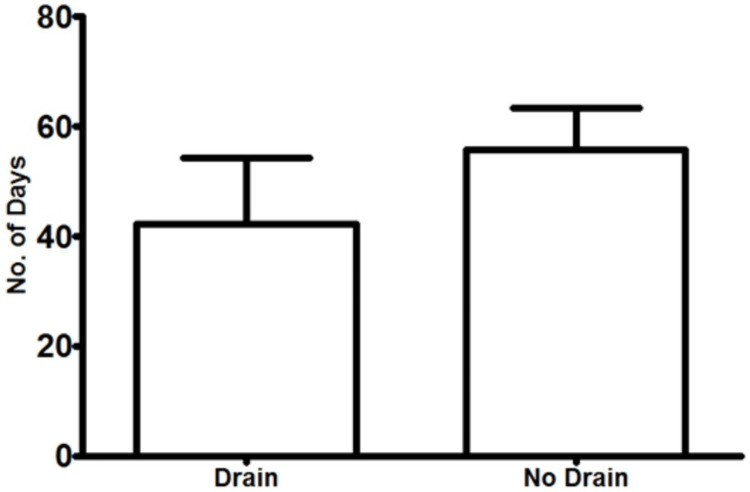
Time to wound healing in the CB versus CBD group. The presence of a drain did not significantly affect time to wound healing in patients with drains versus those without: 55.8 days versus 42.3 days, respectively, p = 0.3648. CB = compression bandage alone; CBD = compression bandage with drain

The mean hospital stay in the CB group was significantly lower than that of the CBD group (4.98 days, SD = 8.574 vs. 9.84 days, SD = 7.647, respectively, p = 0.0125) (Figure 1). The incidence of the other variables between the CB and CBD groups was similar, including SSI (21.3% (16/75) vs. 24.0% (6/25), p = 0.7804) (Figure 2), seroma (25.0% (19/76) vs. 36.0% (9/25), p = 0.2865) (Figure 3), haematoma (0.026% (2/76) vs. 8.0% (2/25), p = 0.2325) (Figure 4), and time to wound healing (55.8 days, SD = 63.59 vs. 42.3 days, SD = 58.88, p = 0.3648) (Figure 5). Missing or ambiguous missing data were omitted.

CB patients had lower mean length (62.8 mm vs. 88.3 mm, p = 0.447), width (43.4 mm vs. 64.9 mm, p = 0.207), and depth (52.5 mm vs. 79.7 mm, p = 0.114) compared to CBD patients, but no statistical significance was observed (Table 1).

**Table 1:** Tumour dimensions of CB versus CBD patients. CB = compression bandage alone; CBD = compression bandage with drain; CI = confidence interval

Dimension	n (CB)	n (CBD)	Mean CB (95% CI)	Mean CBD (95% CI)	P-value
Length	60	19	62.8 (51.2-74.3)	88.3 (56.9-119.8)	0.447
Width	60	19	43.4 (36.7-50.1)	64.9 (43.9-86.0)	0.207
Depth	52	17	52.5 (40.9-64.2)	79.7 (58.3-101)	0.114

A total of 106 patients from a regional STT specialist service were analysed. Data were collected on patient demographics (Table 2), tumour type as confirmed histologically (Table 3), tumour location (Table 4), prognosis (Table 5), and type of surgery (Table 6).

**Table 2:** Demographics of limb STT patients. STT = soft-tissue tumour

Patient demographics	n
Total patients	106
Male	63
Female	43
Age (years)	
Median age	57
Range	16–97

**Table 3:** Type of tumour in limb STT patients. STT = soft-tissue tumour

Tumour type	n
Myxofibrosarcoma	21
Myxoid liposarcoma	18
Undifferentiated pleomorphic sarcoma	12
Leiomyosarcoma	11
Spindle cell sarcoma	8
Synovial sarcoma	7
Fibromyxoid sarcoma	4
Malignant peripheral nerve sheath tumour	4
Angiosarcoma	4
Dedifferentiated liposarcoma	3
Myxoinflammatory fibroblastic sarcoma	2
Malignant perivascular epithelioid tumour	2
Epithelioid sarcoma	2
Fibrosarcoma	1
Myxoid chondrosarcoma	1
Pleomorphic liposarcoma	1
Fibroblastic sarcoma	1
Kaposi’s sarcoma	1
Mesenchymal chondrosarcoma	1
Clear cell sarcoma	1
Neurothekeoma	1

**Table 4:** Location of tumours in limb STT patients. STT = soft-tissue tumour

Tumour location	n
Thigh	47
Leg	20
Arm	17
Groin	5
Knee	4
Shoulder	4
Hand	3
Foot	2
Elbow	2
Axilla	1
Chest	1

**Table 5:** Prognosis in limb STT patients. STT = soft-tissue tumour

Outcome	n
Metastasis	19
Recurrence	8
Deceased	12

**Table 6:** Type of surgery used in our cohort of patients.

Type of surgery	n
Excision/resection	96
Amputation	10

## Discussion

Limb-sparing resection, with or without adjuvant multimodality therapies, is the ideal treatment for STT. Postoperative local non-oncologic wound complications are common and typically occur within six weeks of surgery [5]. Such complications include SSI, seroma, haematoma, and wound dehiscence, all of which can directly and indirectly adversely affect morbidity and mortality. Various risk factors affecting wound complication incidence have been documented, including tumour location, neoadjuvant chemotherapy, and radiotherapy [6]. When poorly managed, these complications may necessitate limb amputation. As such, various preventative measures are taken during and after the procedure, including close attention to haemostasis using Surgicel, closure of dead space as much as possible, and insertion of surgical drains. Adequate haemostatic control and closure of dead space are well documented as factors influencing postoperative complications [3-7]. However, despite abundant usage, there is no detailed study, to our knowledge, evaluating the effect of drains on postoperative morbidity in STT surgery. Aho et al. reported that placement of a surgical drain did not affect the rate of seroma formation (25% vs. 19%, p = 0.39) in STT resection [3], and meta-analyses of subcutaneous surgical wound drainage and subcutaneous drainage after caesarean delivery yielded similar results [8,9]. Another study also reported that the use of drains did not noticeably affect the incidence of wound complications in STT resection, although no statistical analysis was performed [5].

The mean hospital stay was the only variable to show statistical significance between the two groups (4.9 days vs. 9.8 days in the CB and CBD, respectively, p = 0.0125). This is likely attributed to the fact that CBD patients remain in the hospital until satisfactory drainage is achieved. This is typically 50 mL/24 hours, although this can vary between surgeons. Drains were typically removed within 10 days. All other tested variables, including time to wound healing, SSI, and formation of seroma and haematoma, showed no statistical significance. Although wound dehiscence is a well-recognised complication of STT resection [6], it was not included in the final analysis, as a primary cause is SSI, which was another variable. Differentiating between dehiscence secondary to SSI versus other causes was unreliable, and, therefore, determining the additional effect of surgical drain insertion was difficult.

Our findings suggest that surgical drains are of no benefit following limb STT resection. In fact, the senior author has not been using drains for over 10 years. Meticulous haemostasis via Surgicel, sufficient dead space closure, and compression bandage alone may suffice in mitigating the effects of and preventing postoperative complications. The addition of a drain may only prolong hospital stays without reducing postoperative morbidity. This suggests that drains may cause more harm than good given the risk of cross-infection, such as chest or urinary tract infections, with an increased hospital stay. Although some centres are known to discharge patients with drains in-situ with a view to outpatient review, the senior author and other tumour surgeons at our centre preferentially kept patients in the hospital for monitoring until under 50 mL/hour was drained. This is due to the logistical difficulties associated with regular drain changes outside of the hospital, as well as the risk of patients dislodging drains and the potential resultant risk of infection.

Preoperative radiotherapy at a dose of 50 Gray was routinely given for all deep-seated tumours. Although radiotherapy is known to increase the likelihood of postoperative wound complications including SSI, this was administered in a different centre, and individual patient data regarding radiotherapy was not always obtainable. As such, preoperative radiotherapy was not included in the analysis.

Our centre utilises paper records for detailed events regarding inpatient stay, whereas electronic records typically only mention a summary of events. Length of hospital stay was recorded rather than length of time until medically fit for discharge as not all patient paper records could be obtained, although once criteria for drain removal were satisfied, patients were generally deemed medically fit for discharge. Therefore, factors other than drain in-situ, including logistics of social care, may have contributed to prolonged hospital stays, as not all electronic records had this accurately documented.

Patients were treated with CB versus CBD as per surgeon preference. Although patients selected for CBD had higher mean length (88.3 mm vs. 62.8 mm, p = 0.447), width (64.9 mm vs. 43.4 mm, p = 0.207), and depth (79.7 mm vs. 52.5 mm, p = 0.114) compared to CB, no statistical significance was demonstrated. Radiological reports occasionally included only one or two measured dimensions. A total of 27 STT patients had no recorded length or width of the tumour, and 37 STT patients had no documented depth. These data were, therefore, omitted from statistical analysis. The precise location of tumours, including relation to fascia, was only occasionally documented, and this was also not included in the final analysis, although deeper lesions are known to have higher rates of postoperative seroma or haematoma formation.

The incidence of SSI was similar in the CB versus CBD groups (21.3% vs. 24.0%, respectively). This was similar to SSI rates reported in a separate study (18.4%) in postoperative STT patients, although the presence of a drain was not included as a variable [10].

The incidence of haematoma was very low in both the CB and CBD groups. This matches a previous study which reported four haematomas in 118 STT patients [6]. Therefore, another study with greater numbers of postoperative haematomas may be needed to accurately evaluate the effect of surgical drains.

Interestingly, our results on seroma formation were similar to those reported in other studies [3,5]. Seroma develops after STT resection in the dead space left behind by the tumour. In theory, if this dead space can be completely obliterated, there will be no available cavity for serous fluid to collect, thereby rendering drains unnecessary. However, in particularly large tumours, total eradication of dead space is not always possible, and drains may remain useful. A larger, prospective trial is needed for further evaluation.

A total of 96 patients underwent radical or local excision (margins of 1-2 cm where possible), of whom 24 had drains, and 10 underwent amputation, of whom one had a drain. The level of amputation depended on the tumour location and included above/below knee, above elbow, and mid-forearm amputations. Due to the small number of CBD patients who underwent amputation, statistical analyses could not be performed. Further studies with larger patient numbers are needed.

As per local policy, lower limb STT patients received routine prophylactic anticoagulation for two weeks via low-molecular-weight heparin (dalteparin), whereas upper limb STT patients were on a case-by-case basis as per risk-benefit. Groin and axilla STT patients received one dose of prophylactic intravenous co-amoxiclav prior to the surgery. Groin wound compression was achieved via Gamgee Tissue and Mefix dressings. Tranexamic acid was not used, and no deep vein thrombosis or pulmonary embolism was recorded.

Limitations

As would be expected of a single-centre cohort, the study comprised a small, heterogeneous population. Radiological reports with measured dimensions were not available for all patients in this study. A total of 27 STT patients had no recorded length or width of the tumour, and 37 STT patients had no documented depth. Because preoperative radiotherapy data were only occasionally available, this was not factored into the analysis. Locations of STTs in relation to surrounding structures or fascia also could not be recorded due to missing data. Unobtainable paper records and condensed electronic records meant that the length of hospital stay was recorded rather than the length of time until medically fit for discharge, and, as such, factors other than a drain in-situ may have contributed to prolonged hospital stays.

## Conclusions

There is evidence to suggest that the use of surgical drains following limb STT surgery lengthens hospital stay and otherwise has little effect on postoperative morbidity. However, in large tumours where dead space cannot be fully eradicated, drains may be effective. A prospective trial with larger numbers of patients stratified by tumour location and neoadjuvant therapies is needed to confirm these findings.
